# Intracellular Bacterial Symbionts in Corals: Challenges and Future Directions

**DOI:** 10.3390/microorganisms9112209

**Published:** 2021-10-23

**Authors:** Justin Maire, Linda L. Blackall, Madeleine J. H. van Oppen

**Affiliations:** 1 School of Biosciences, The University of Melbourne, Melbourne, VIC 3010, Australia; linda.blackall@unimelb.edu.au (L.L.B.); madeleine.van@unimelb.edu.au (M.J.H.v.O.); 2 Australian Institute of Marine Science, Townsville, QLD 4810, Australia

**Keywords:** symbiosis, coral, intracellular, bacteria

## Abstract

Corals are the main primary producers of coral reefs and build the three-dimensional reef structure that provides habitat to more than 25% of all marine eukaryotes. They harbor a complex consortium of microorganisms, including bacteria, archaea, fungi, viruses, and protists, which they rely on for their survival. The symbiosis between corals and bacteria is poorly studied, and their symbiotic relationships with intracellular bacteria are only just beginning to be acknowledged. In this review, we emphasize the importance of characterizing intracellular bacteria associated with corals and explore how successful approaches used to study such microorganisms in other systems could be adapted for research on corals. We propose a framework for the description, identification, and functional characterization of coral-associated intracellular bacterial symbionts. Finally, we highlight the possible value of intracellular bacteria in microbiome manipulation and mitigating coral bleaching.

## 1. Introduction

Symbiosis, the association between two or more organisms of distinct species [1,2], is ubiquitous in nature. It includes a diversity of associations ranging from parasitism to mutualism [3,4,5,6,7]. Mutualistic interactions are believed to be among the main evolutionary driving forces, as they generate diversity and accelerate species adaptation via the creation of biological novelty [8,9,10]. Symbiosis is found in all ecological niches and at different levels of host integration. This includes ectosymbioses (extra-organismal symbioses) and endosymbiosis (intra-organismal symbioses, which can be either extracellular, e.g., in a host cavity or between cells, or intracellular) [11]. Intracellular symbioses are the most intimate forms of symbiosis, often leading to complete inter-dependence between the different partners [12]. It is now widely accepted that eukaryotic organelles, mitochondria and plastids, originated from endosymbiotic integration, highlighting the most extreme level of integration between host and endosymbiont [13].

Intracellular symbiosis between eukaryotes and bacteria is widespread across the tree of life and is known from insects [14,15], marine organisms such as bivalves and tubeworms [16,17], plants [18,19], and protists [20]. The intracellular nature of these associations provides multiple advantages. First, metabolic exchanges are optimized by host–endosymbiont proximity. For example, in the pea aphid *Acyrtosiphon pisum*, cells harboring the endosymbiont *Buchnera aphidicola* upregulate the expression of genes involved in the transport of cationic amino acids, the very same amino acids provided by *B. aphidicola* to its aphid host [21]. Intracellular habitats provide endosymbionts with a stable, nutrient-rich environment, although this has historically been hard to demonstrate [22,23]. Second, by sequestering endosymbionts within cells, host immune homeostasis is maintained by preventing unnecessary and potentially harmful immune responses that could be triggered by endosymbionts. This was shown in the cereal weevil *Sitophilus zeamais*: while the endosymbiont *Sodalis pierantonius* can produce peptidoglycan and can trigger a host immune response [24,25], immune homeostasis is preserved through the high production of host enzymes able to cleave peptidoglycan, thus rendering it non-immunogenic [24]. Finally, population control is also eased in an intracellular context, as was described in symbioses between legume plants and nitrogen-fixing bacteria of the genus *Rhizobium*. *Rhizobium* are environmentally acquired by their host and trigger the formation of root nodules in which they are intracellularly housed [18]. Oxygen provision by the plant host is decreased in nodules housing less metabolically efficient strains, leading to lower bacterial density and smaller nodules [26].

**Figure 1:**
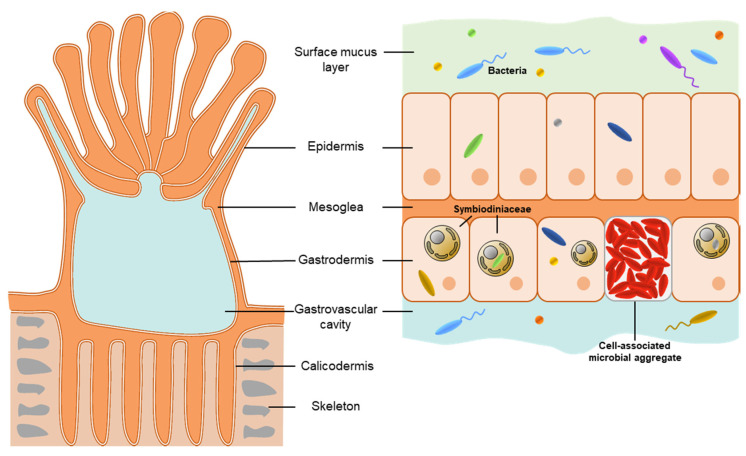
Coral anatomy and microbial associations. A coral colony is composed of many identical polyps (left panel shows an individual polyp), connected by interlaying tissues and sharing a calcium-carbonate skeleton. Each polyp is composed of two cellular layers, the epidermis and the gastrodermis, separated by a largely acellular layer, the mesoglea, although it contains fibroblasts and amoebocytes [55]. The epidermis is covered by the surface mucus layer, where bacteria are highly abundant [42]. Symbiodiniaceae reside in the gastrodermis but are found in the mucus as well [56,57]. Intracellular bacteria and cell-associated microbial aggregates can occur in both cellular layers. Symbiodiniaceae cells have also been reported to harbor intracellular bacteria [51]. Other microorganisms, including viruses [58], archaea [59], fungi [60], and other eukaryotes [61,62], associate with corals and are not represented in this figure.

Scleractinian corals rely on intracellular photosynthetic dinoflagellates of the Symbiodiniaceae family for their health and survival [27,28,29], which they house within their gastrodermal cells (Figure 1). In exchange for shelter, inorganic carbon, and other coral metabolic products, Symbiodiniaceae translocate photosynthate directly into the coral cells, thus providing their coral host with an organic carbon source [30,31]. This intracellular photosymbiosis is essential for the survival of scleractinian corals and enables them to build the three-dimensional structures of coral reefs, which are estimated to be home to more than 25% of the multicellular eukaryotic species in the global oceans [32,33]. While the coral–Symbiodiniaceae symbiosis has survived millions of years and episodes of mass extinctions [29], it has proved fragile in the face of the current climate crisis: the breakdown of this association—coral bleaching—often results in coral starvation, increased chances of opportunistic infection, and eventually death [34,35]. Coral bleaching is believed to be caused by excessive production of reactive oxygen species (ROS) by Symbiodiniaceae under thermal and light stress, leading to their death or expulsion from the coral tissues [36,37,38]. Climate change-related seawater temperature increases have caused a surge in mass coral bleaching events in the past decade and have led to an unprecedented global deterioration of coral reefs [39].

Corals also associate with a multitude of bacteria, which are receiving a rapidly increasing amount of attention [40]. Coral-associated bacteria are thought to be involved in metabolic functions, such as carbon, nitrogen, and sulfur cycling, as well as host protection from pathogens [40,41]. Bacteria have been detected in every coral microhabitat, including the mucus [42]; tissue layers [43,44]; gastrovascular cavity [45]; skeleton [46]; and in the case of cyanobacterial pathogens, the mesoglea [47]. However, intracellular coral-associated bacteria, specifically, remain under-studied, with only a handful of reports describing intracellular bacterial symbionts [43,48,49,50,51]. In this review, we outline the knowledge gaps in the study of intracellular bacterial symbiosis in corals and provide directions to this important field of study by drawing on examples from other animal and plant systems that have been successful in the study of intracellular bacterial symbioses. We also provide examples of applications of intracellular bacteria in the microbiome manipulation field, which is increasingly brought forward as an approach to mitigate coral bleaching [52,53,54].

## 2. The Search for Intracellular Bacteria

**Figure 2:**
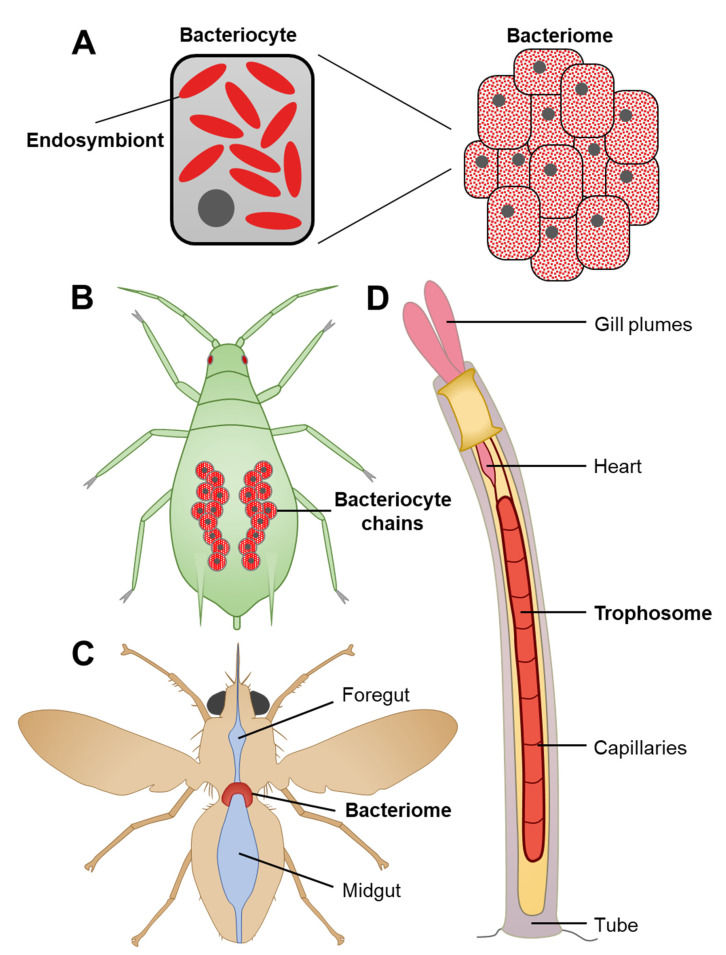
Morphological diversity of symbiotic structures. In organisms such as insects, high densities of endosymbionts are housed in specialized host cells, called bacteriocytes, that sometimes form a dedicated organ, the bacteriome (**A**). In the pea aphid *A. pisum*, giant bacteriocytes (diameter > 100 µm in adults) are grouped in two abdominal chains (**B**) [14]. They house *B. aphidicola*, which provides most essential amino acids to its sap-feeding host [63]. In the tsetse fly *Glossina morsitans*, bacteriocytes form a bacteriome around the gut (**C**) [67]. Its endosymbiont *Wigglesworthia glossinidia* synthesizes B vitamins that are absent in the fly’s hematophagous diet [68]. In the tube worm *Riftia pachyptila*, bacteriocytes form a trophosome that is directly linked to the circulatory system (**D**). This tubeworm species usually lives near hydrothermal vents. Carbon dioxide and hydrogen sulfide are internalized by the plume gills and transferred through the circulatory system to the trophosome, where chemosynthetic endosymbionts are able to metabolize those dissolved gases and to provide organic matter to their host [16].

Fifty-five years ago, Paul Buchner provided one of the most comprehensive bodies of work describing endosymbiosis in insects as well as in a few mollusks and fishes, using only light microscopy [14]. This seminal study was conducted long before electron microscopy, fluorescence microscopy, and DNA sequencing were accessible. Despite the availability of such modern techniques, observations of intracellular bacteria are still scarce in cnidarians. Certain intracellular bacteria were observed with fluorescent microscopy (via fluorescence in situ hybridization (FISH)) and electron microscopy in the epithelial cells of *Montastraea cavernosa* [48]; in the gastrodermal cells of *Acropora granulosa* [43], *Acropora cervicornis*, *Acropora hyacinthus,* and *Acropora cytherea* [49,50]; and in Symbiodiniaceae cells, both in culture and freshly isolated from the coral *Galaxea faciscularis* and the sea anemone *Exaiptasia diaphana* [51]. However, no bacteria-housing structures have been described in corals so far, which has made in situ localization challenging as individual bacteria or bacterial clusters might not be detected by conventional methods or could easily be regarded as artefacts. By contrast, most insects house their endosymbionts in specialized cells, called bacteriocytes, that sometimes group together in an organ, the bacteriome [14,63] (Figure 2A). Within bacteriocytes, bacterial density is very high, as these cells are usually filled with endosymbionts, hence being highly visible even with light microscopy. This ‘compartmentalization’ is believed to have evolved independently in many insect lineages, as it is found in widely divergent orders and exhibits great morphological variability (Figure 2B–C). Similar specialized, endosymbiont-filled cells have also been described in platyhelminths [64] and annelids [65], in an organ called the trophosome (Figure 2D). Interestingly, it was shown in tubeworms that the trophosome originates from mesodermal tissue [66]. The absence of a mesodermal tissue layer during cnidarian embryonic development, which possess the largely acellular mesoglea layer instead, could explain the absence of specialized morphological structures that house endosymbionts.

Nonetheless, in a wide range of coral species, structures called cell-associated microbial aggregates (CAMAs) have been observed, both in the epidermis and the gastrodermis [44,69,70,71,72,73,74,75] (Figure 1). In the coral *A. hyacinthus*, CAMAs were present in all observed tissues: tentacles, actinopharynx, mesentery, mesenterial filaments, coenenchyma, and calicodermis [70]. In *Porites compressa*, transmission electron microscopy revealed that CAMAs were sometimes surrounded by what appeared to be a membrane, suggesting an intracellular location [72]. Similarly, in the cnidarian model, the sea anemone *Exaiptasia diaphana*, CAMAs observed in tentacles seemed to be contained within vacuoles, which were intracellular when the CAMA was small and extracellular when the CAMA was big (i.e., bigger than a regular anemone cell) [76]. The possible intracellular nature of these aggregates therefore makes them of great interest; however, there is little known besides their description and localization. More detailed work, especially electron microscopy and DNA sequencing, should be undertaken to determine the exact subcellular location and taxonomic affiliation of these aggregates. The mechanisms behind their formation are also unknown and should be investigated, as it could involve bacterial signals (e.g., quorum-sensing and chemotaxis), host signals, or a combination of both. In the legume–*Rhizobium* symbiosis [18], *Rhizobium* present in the soil are attracted by flavonoids produced by the host plant and released in the environment. In response, *Rhizobium* secretes Nod factors that are recognized by the host plant and trigger signaling cascades leading to the formation of root hair curls that essentially trap *Rhizobium*. From there, *Rhizobium* move towards the plant root cells, infect them, and finally trigger the formation of specialized nodules, a structure that bears resemblance with CAMAs. Similarly, chitin and chitobiose were identified as chemoattractants in the association between the squid *Euprymna scolopes* and the luminescent extracellular bacterium *Vibrio fischeri* [77]. Indeed, a gradient of chitobiose lures environmental *V. fischeri* into the squid [77], where it triggers the maturation of a so-called light organ [78], although it remains extracellular. Cells of *Endozoicomonas montiporae*, isolated from *Montipora aequituberculata*, formed aggregates in vitro when provided with dimethylsulfoniopropionate (DMSP) as a carbon source, forming structures similar to CAMAs [79]. This indicates that metabolites potentially originating from the coral host and/or Symbiodiniaceae could initiate CAMA formation.

Symbiodiniaceae cells are also a microhabitat of high interest, as algae–bacteria interactions have been widely described (Box 1). In fact, Symbiodiniaceae-associated bacteria were recently shown to be abundant, both extracellularly and intracellularly, in cultured Symbiodiniaceae and in Symbiodiniaceae freshly isolated from two cnidarians [51]. Intracellular bacterial communities showed great similarity across Symbiodiniaceae species, suggesting they might have conserved functions in these algae [51]. Additionally, the phycosphere of free-living Symbiodiniaceae, the extracellular layer around the cell composed of algal exudates such as organic carbon, may attract and support bacterial metabolism and growth [80,81,82]. If similar mechanisms occur *in hospite*, these might attract bacteria into the gastrodermal cells. As Symbiodiniaceae cells are of a similar size to their host cells, bacteria entering gastrodermal cells would be in immediate contact with Symbiodiniaceae, hence facilitating interactions. Some metabolites are expected to cross the multiple symbiosomal membranes and to facilitate Symbiodiniaceae-bacteria communication; however, such metabolites and the mechanisms behind their exchange between Symbiodiniaceae and bacteria remain uninvestigated. In line with this, cyanobacteria-like structures were shown to be abundant in gastrodermal cells and in close proximity with Symbiodiniaceae in *A. hyacinthus* and *A. cytherea* [50]. Symbiodiniaceae–bacteria interactions have seen growing interest in recent years and have been suggested as a potentially critical factor in Symbiodiniaceae—and in turn coral—health [83,84,85,86] (Box 1), but any proven functions of intracellular bacteria housed in Symbiodiniaceae, either in culture or *in hospite*, that could be interpreted as mutualistic remain to be determined.

Box 1 **Algae–bacteria interactions.**

Interactions between bacteria and microalgae have been widely studied [87,88]. For example, vitamin B12 synthesis is dependent on bacteria in green algae [89,90], red algae [91], and dinoflagellates [91]. The use of axenic algal cultures, thereafter supplemented with vitamin B12-producing bacteria (e.g., *Halomonas* sp. [91]), or vitamin B12 directly, was instrumental in reaching those conclusions. Bacteria also promote iron assimilation in dinoflagellates [92] and growth through hormone production in diatoms [93]. While most of these interactions are expected to happen extracellularly, in the phycosphere [80,81], transmission electron microscopy and confocal laser scanning microscopy have enabled the detection of intracellular bacteria in a wide range of microalgae [94,95,96,97,98]. Hence, interactions between intracellular bacteria and Symbiodiniaceae are expected, both within coral gastrodermal cells and within Symbiodiniaceae cells. The field of Symbiodiniaceae–bacteria interactions is still in its infancy [84], although several recent papers have investigated the taxonomy and potential functions of bacteria associated with *ex hospite* Symbiodiniaceae.

The composition of bacterial communities was shown to differ between Symbiodiniaceae strains, although a few taxa were consistently detected, including *Labrenzia, Marinobacter, Muricauda, Hyphomicrobium,* and *Methylobacterium* [51,85,86]. Community composition was shown to change following exposure to experimental thermal stress, but these modifications were less marked in the thermally tolerant *Durusdinium trenchii* [85], suggesting that Symbiodiniaceae–bacteria interactions could play a role in Symbiodiniaceae’s thermal resilience. Incidentally, a *Muricauda* strain was recently shown to provide a ROS-scavenging carotenoid, zeaxanthin, to cultured *Durusdinium* sp., thereby participating in its resistance to thermal and light stress [83]. Furthermore, two studies have described Symbiodiniaceae–bacteria interactions related to sulfur cycling. Experiments using nanoscale secondary ion mass spectrometry (NanoSIMS) have revealed that DMSP produced by cultured Symbiodiniaceae (*Cladocopium goreaui*) was taken up by *Pseudovibrio* spp. and metabolized into dimethylsulfate [99]. More recently, it was shown that DMSP degradation by bacteria was stimulated when in close proximity to DMSP-producing Symbiodiniaceae [100]. DMSP and its breakdown products have ROS-scavenging abilities and could be involved in excess-ROS scavenging during coral bleaching events [101]. Hence, evidence towards a role for bacteria in Symbiodiniaceae’s thermal resistance and, potentially, coral bleaching is accumulating, yet *in hospite* data remain rare, with only one study so far highlighting metabolic Symbiodiniaceae–bacteria interactions within a cnidarian holobiont. NanoSIMS analysis showed that nitrogen was translocated from *Vibrio alginolyticus* and *Alteromonas* sp., prelabeled with ^15^N-labeled ammonium, to Symbiodiniaceae within the coral *Pocillopora damicornis* [102]. However, in both *in hospite* and *ex hospite* studies, bacteria were either extracellular [99,100] or their localization was not investigated.

Thus, considering the wide variety of algae–bacteria interactions described so far, the complexity of the bacterial communities associated with *ex hospite* Symbiodiniaceae, and the preliminary evidence that they could be involved in Symbiodiniaceae’s performance under thermal stress, *in hospite* Symbiodiniaceae–bacteria interactions, both in gastrodermal cells and in Symbiodiniaceae cells, should be deeply investigated.

Finally, as mutualistic intracellular bacteria are likely to be vertically transmitted from parent to offspring [12], coral gonads and gametes should be studied for the presence of intracellular bacteria as well. Corals possess two modes of sexual reproduction: broadcast spawning and brooding. Broadcasters release eggs and sperm into the water column for external fertilization and development; brooders release sperm that internally fertilize eggs within polyps, and brood larvae until maturity [103]. Coral gonads—spermeries and ovaries—form anew at each reproductive cycle in the endodermal mesenteries [104]. In insects, endosymbiont vertical transmission is often achieved through a persistent bacterial population present in the reproductive organs, often in females ([67,105,106,107,108], but see [109] for a case of paternal transmission). Endosymbionts often infect the egg during oogenesis [63,105,108] or are deposited on the egg after oviposition via capsules or jellies [110]. As coral gonads are transient, it is unlikely that they would possess a persistent bacterial population able to infect gametes. Nonetheless, the mesentery portions where gonad formation and gametogenesis occur should be investigated through FISH and 16S rRNA gene metabarcoding for the presence of bacteria that might be vertically transmitted through gametes. Several 16S rRNA gene metabarcoding studies in broadcasters have shown the presence of shared bacteria between adults and gametes/early life developmental stages [111,112], suggesting the vertical transmission of some bacterial associates. However, FISH experiments have thus far not detected the presence of bacteria in the gametes of broadcasters [111,112], prompting the hypothesis that bacteria might be transferred through mucus present on the surface of egg–sperm bundles [113], which would be lost during classical FISH fixation procedures with aqueous paraformaldehyde. Water-free fixation methods, such as Carnoy’s solution [114], should be employed to preserve the mucus and to test this hypothesis. Interestingly, both 16S rRNA gene metabarcoding and FISH experiments revealed vertical transmission of bacteria in larvae of the asexual brooder *Pocillopora acuta* [71]. The situation is reminiscent of the tsetse fly *G. morsitans*, which also broods larvae one at a time until it reaches maturity and transmits its primary endosymbiont *W. glossinidius* through milk secretions that feed the developing larvae [115]. Symbionts might be fed and vertically transmitted in a similar way to developing planulae in brooding corals.

## 3. The Identification of Intracellular Bacteria

The identification of intracellular bacteria has always been a challenging task. Due to their intracellular lifestyle and potentially high level of dependence on their hosts, most endosymbionts are not pure-culturable. Indeed, some vertically transmitted endosymbionts are known to have undergone massive genome shrinkage (Box 2). Endosymbiont genes encoding metabolic pathways that are redundant when *in hospite* are often lost, leading to complete dependency on the host for proper functioning and survival [12]. Culturing procedures that require cell or tissue passaging have proved successful. For example, Chlamydiae and *Mycobacterium leprae* depend on host cells to replicate and are successfully grown in the lab in amoebae and armadillos, respectively [116,117]. Culture-independent techniques, such as 16S rRNA gene metabarcoding, have also been widely applied to identify coral-associated bacteria [118]. However, extracellular and intracellular bacteria cannot be distinguished in metabarcoding analyses, unless their taxonomic affiliation places them in known intracellular taxa. For example, *Candidatus* Aquarickettsia rowheri, a novel member of the Rickettsiales order (known to be obligate intracellular bacteria), have been found to widely associate with corals [49]. However, this bacterium has been linked to coral dysbiosis as it is thought to thrive off excess inorganic nitrogen, subsequently taking up host resources, slowing coral growth and increasing disease susceptibility and mortality [49,119,120]. Unlike most mutualistic endosymbionts, *Ca*. A. rowheri is not vertically transmitted [121], although it is not uncommon for intracellular pathogens to spread horizontally [122]. Simkaniaceae, belonging to the intracellular Chlamydiales order, have also been reported in high abundances in several coral [112,123,124,125] and Symbiodiniaceae species [51], although these bacteria might be associated with protists present in the samples. Endosymbiont identification in insects has been largely facilitated by their typically very low diversity, with one bacterial species usually being exclusively associated with one insect species [126] and by them being condensed in bacteriocytes and bacteriomes. Crude extracts of bacteriomes have often been enough to identify the single bacterial species housed in this organ. To identify bacteria that are likely important for holobiont functioning and health, a ‘coral core microbiome’ approach was recently proposed to identify bacteria that persist across samples (e.g., of a same coral species, a same reef, etc.) [127]. However, all mutualistic species in a holobiont are present due to not only their taxonomic identity but also their functional roles. Different holobionts might house different bacterial taxa that provide the same functions in every holobiont. To avoid missing potentially important functions supported by bacteria of different taxonomic affiliations, a better concept would therefore be the ‘core microbiome function’.

Box 2 **Genome evolution in bacterial endosymbionts.**

The intracellular lifestyle and vertical transmission of endosymbionts across successive generations imposes strong evolutionary constraints. On the one hand, contact with the environment is restricted, hence limiting the possibility of genetic recombination and horizontal gene transfers with environmental bacteria. On the other hand, only a small subset of symbionts is vertically transmitted each generation, thus creating an evolutionary bottleneck leading to genetic drift and increased mutational fixation rate. Furthermore, their strictly intra-organismal status provides a stable environment, which relaxes selection pressure on many biological functions. Eventually, this leads to massive gene pseudogenization and deletion [12,128,129]. Loss of DNA-repair-encoding genes also amplifies pseudogenization [130,131,132], and subsequent defects in genome reparation lead to a genome composition bias in favor of the less energy-consuming AT nucleic bases [128,129,132]. Over long co-evolutionary periods, massive gene deletion leads to drastic genome shrinkage [12,126,133].

To date, the smallest genome of an endosymbiont is that of *Nasuia deltocephalinicola*, the endosymbiont of the leafhopper *Macrosteles quadrilineatus.* It is composed of only 112 kb [128], substantially smaller than, for instance, the ~4 Mb genome of the free-living bacterium *Escherichia coli* [134]. This phenomenon is, however, not restricted to intracellular symbionts, with the notable example of the 271 kb genome of *Stammera*, the vertically transmitted extracellular symbiont of the beetle *Cassida rubiginosa* [107]. Symbionts possessing an environmental phase can also display some degree of genome shrinkage, although generally not to the same extent as symbionts with strict vertical transmission [110]. An example is the two extracellular obligate symbionts of the flashlight fish *Anomalops katoptron*, which can be expelled in the seawater by their hosts and can survive in the environment [135]. The genomes of these symbionts are 1 Mb in size. Their high gene content and low pseudogene abundance suggest genomic stasis [136], albeit at a much larger size than vertically transmitted bacteria. They have also retained most genes necessary for DNA recombination, a feature that is absent from vertically transmitted obligate endosymbionts [136].

A compelling pattern of bacterial genome degeneration is the loss of metabolic genes that show redundancy with host functions. For example, the only metabolic function retained by the 226 kb genome of *Nardonella*, an endosymbiont of the weevil *Pachyryhnchus infernalis*, is the ability to synthesize tyrosine, which its host cannot synthesize or obtain from its diet [137]. All other metabolic functions are assumed to be undertaken by the host. This pattern is widespread across nutritional endosymbionts, in which the metabolic functions are usually complementary to those of their host, and leads to host-symbiont metabolic co-dependence [12]. This co-dependence and the difficulty to recreate artificial media that properly mimic intracellular conditions are the main barriers to culturing obligate intracellular and/or vertically transmitted symbionts.

Thus, we recommend applying the ‘core microbiome’ approach to the identification of intracellular bacteria that stably associate with corals, as widespread intracellular symbionts are more likely to bear significant functions. In order to eliminate extracellular bacteria and have the ability to link putative bacterial functions with their tissue of origin, organs or tissue layers must be studied independently from one another. Laser capture microdissection (LCM) has recently emerged as a tool enabling such studies, allowing for the separation of groups of cells. Ainsworth et al. (2015) applied this technique to identify bacteria associated with gastrodermal cells (i.e., cells also harboring Symbiodiniaceae), that are therefore putatively intracellular [43]. It was concluded that bacterial communities in the gastroderm differed significantly from those of the whole coral and that abundant, core intracellular bacteria, *Ralstonia* sp. and *Cutibacterium* sp. (formerly *Propionibacterium* sp.), were under-represented in whole coral communities, confirming the need to study bacterial communities at the sub-organismal level [43]. However, both *Ralstonia* and *Cutibacterium* are commonly found in negative controls and reported as contaminants [138,139], and could be over-represented in low-biomass samples, such as samples from LCM experiments. As negative controls were not reported [43], it remains unknown whether *Ralstonia* and *Cutibacterium* are contaminants or true intracellular bacteria. This same study also explored the microbiome of tissue layers only, i.e., the gastroderm, the epithelium, and the mesoglea, although LCM was not used to separate these tissues, and surface or interstitial bacteria could have contaminated these samples. Nonetheless, separating cells from specific microhabitats, through LCM or even manual dissection when possible, should be encouraged in the future to identify intracellular bacteria and to discriminate them from extracellular bacteria.

Finally, once presumed intracellular bacteria are identified, their long-term presence in an organism and in related species must be assessed, as endosymbionts that persist over time and under changing environments are more likely to bear biological and ecological significance. For example, cyanobacteria-like structures were abundantly observed in gastrodermal cells of *A. hyacinthus* and *A. cytherea* both in summer and in winter, indicating a degree of stability in this association [50]. In most insects, endosymbionts are present in 100% of populations, e.g., *Blochmannia* in the carpenter ant *Camponotus* spp. [140] or *Tremblaya* in mealybugs [141], and have been co-evolving with their hosts for millions of years. As such, endosymbionts have been vertically transmitted over the course of many speciation events within insect groups—co-diversification, resulting in phylogenetic congruence or cophylogeny between hosts and endosymbionts. Correlations between the phylogeny of hosts and their associated bacteria have been reported in corals, in particular for tissue- and skeleton-associated bacteria, i.e., closely associated symbionts [142,143,144]. However, as symbiont stability and mode of transmission are mostly unknown, co-evolution cannot be assumed. Hence, future work should be focused on identifying intracellular bacteria, assessing whether they are vertically transmitted and stable throughout their host’s life cycle and evaluating their presence in a wide range of related coral species to examine coral–bacteria co-evolution.

## 4. The Assessment of the Roles of Intracellular Bacteria within the Coral Holobiont

Thus far, proven bacterial functions in corals are mostly related to extracellular bacteria or bacteria of unknown location and studies often relied on culture-dependent techniques. For example, bacteria associated with *Acropora palmata* were shown to produce antibiotics that could potentially protect their coral hosts from pathogens [145]. These bacteria were isolated from coral mucus and are thus extracellular [145]. Similarly, many bacterial functions were inferred from the analysis of whole genomes, which, based on their large sizes, are likely to come from extracellular bacteria [146]. The most compelling evidence of bacterial involvement in nitrogen cycling in corals comes from nanoSIMS data, involving bacterial culturing, stable-isotope (^15^N) labelling, and reinoculation to corals [102,147]. Such experiments are unlikely to work with intracellular bacteria. Nonetheless, a series of in situ experiments have shown (i) the presence of intracellular cyanobacteria in the coral *M. cavernosa* [48]; (ii) their ability to produce nitrogenases, through immunoblotting and immunogold labelling [48]; and (iii) a higher rate of nitrogen fixation, via the acetylene reduction assay, in corals associated with cyanobacteria when compared with corals of the same species lacking cyanobacteria [148]. To our knowledge, this is the only functional study of a coral-associated intracellular bacterium.

A crucial aspect in assessing the function of intracellular, non-pure-culturable bacteria is the possibility of collecting or generating individuals lacking the bacteria, as exemplified by Lesser et al. (2007), who sampled and compared corals of the same species (*M. cavernosa*) and from the same depth, which differed in their association with intracellular cyanobacteria [148]. Comparative analyses of organisms that contain or lack specific intracellular symbionts can thus give insight in the function of the symbiont. This approach has been widely used in insects, particularly with endosymbionts involved in metabolic complementation. For example, the African tick *Ornithodoros moubata* associates with intracellular bacteria of the genus *Francisella* [149]. *Francisella* endosymbionts were removed from the ticks using antibiotics, and these aposymbiotic ticks were reported to have lower nymph emergence rates, lower mass, and physical abnormalities (darker and inflated bodies) [149]. These hampered phenotypes were rescued by the supplementation of the aposymbiotic tick’s diet with B vitamins, a nutrient that is in low abundance in the natural tick’s diet (mammal blood), hence showing that *Francisella* is involved in B vitamin metabolism [149]. However, this approach relies on easily observable differences between symbiotic and aposymbiotic animals, and options to rescue endosymbiont functions (e.g., B vitamins in ticks). Furthermore, antibiotic-driven depletion of endosymbionts in corals, which associate with highly diverse bacterial communities, would also affect extracellular symbionts. An alternative approach may be to try to generate completely axenic organisms and then to re-inoculate them with specific bacteria of interest, although antibiotic treatments have so far had limited success [150].

**Figure 3:**
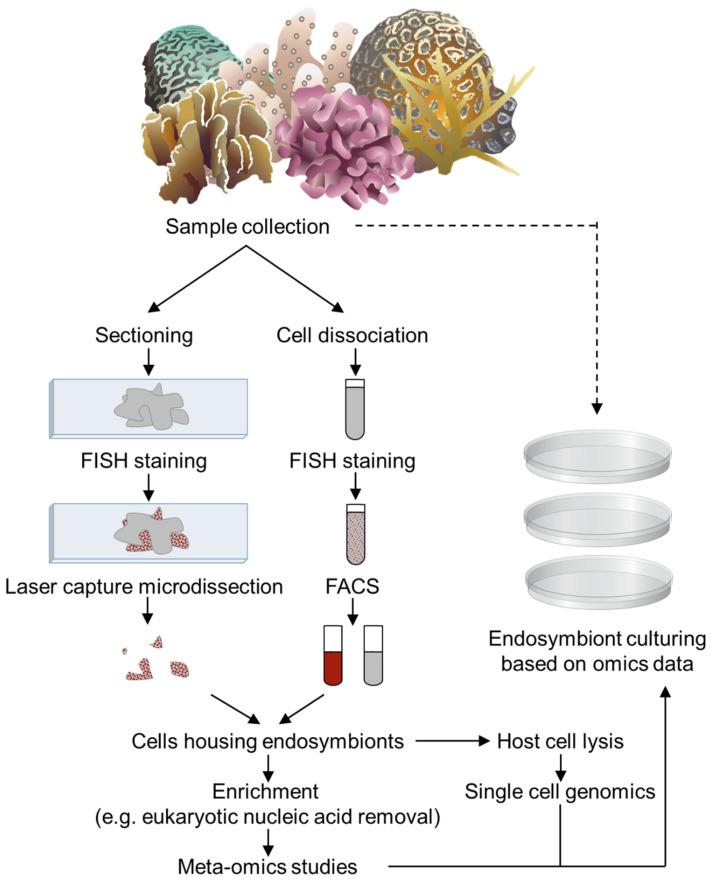
Proposed workflow for meta-omics studies of coral-associated endosymbionts. To optimize meta-omics studies in coral endosymbionts, we recommend the use of techniques allowing for the selection of cells harboring endosymbionts. FISH can be used on tissue sections to highlight endosymbionts, and LCM can allow for the capture of cells harboring them (left). Alternatively, coral cells can be dissociated, stained by FISH, and sorted through FACS (middle) [166]. This would maximize the abundance of bacterial reads in subsequent meta-omics studies. These symbiont-housing coral cells can then be lysed to release intracellular bacteria that can be sorted and individually sequenced through single cell genomics. Furthermore, omics data would give insight into the conditions needed for the culture of coral endosymbionts (right). Coral and plate images courtesy of the Integration and Application Network (ian.umces.edu/symbols/, accessed on 26 May 2020).

With the advent of high throughput sequencing, many tools are now available for specific, functional, *in hospite* studies of non-pure-culturable endosymbionts. The first published genome of a mutualistic endosymbiont was that of *B. aphidicola*, endosymbiont of the pea aphid *A. pisum* [151]. It confirmed the findings of metabolic complementation experiments: *B. aphidicola* can synthesize all essential amino acids and can provide them to its insect host. It also provided a first glimpse into signatures of genome reduction in obligate intracellular bacteria (Box 2), which were confirmed after the publication of dozens of genomes from obligate endosymbionts [126,152]. Assessing such genome degeneration signatures, e.g., reduced size, base composition bias, and high abundance of pseudogenes, in coral-associated bacteria would help in identifying host-dependent and potentially vertically transmitted endosymbionts. Endosymbiont transcriptomics are also becoming more common, with the dual-RNAseq approach, i.e., the simultaneous analysis of host and endosymbiont transcriptomes, gaining traction in recent years [153,154,155,156]. While dual-RNAseq has been applied to study coral–Symbiodiniaceae transcriptomes [157], bacterial transcriptomics are still understudied, mostly because of the high bacterial diversity in corals, the relatively low abundance of bacterial mRNAs compared with eukaryotic mRNAs, and the high costs of rRNA and poly-A tailed mRNA depletion approaches. We must move towards meta-omics to capture the taxonomic, genetic, and functional diversity of intracellular bacteria in corals, ideally coupled with symbiont-containing coral cell enrichment approaches or intracellular bacteria-sorting methods (Figure 3). For example, the genome of the intracellular coral bacterium *Ca*. A. rowheri was obtained through shotgun sequencing of a coral sample, which confirmed its reduced genome size, its inability to produce most amino acids and ATP and thus to replicate on its own, as well as its ability to sense extracellular nitrogen [49]. The latter was hypothesized to participate in the bacterium’s ability to reduce coral health when nitrogen is in excess. Meta-omics data would provide insight into designing suitable culture conditions, including specific metabolites that are needed to obtain pure cultures of coral intracellular symbionts [158]. Obtaining endosymbionts in pure culture could be challenging but not unfeasible. Indeed, a handful of insect endosymbionts have been successfully pure cultured using supplemented media based on their host’s diet [159,160,161,162,163]. For instance, *Sodalis glossidinius*, an intracellular symbiont (although it is also found extracellularly in its host [164]) of the tsetse fly *G. morsitans*, was initially cultured on agar media supplemented with horse blood [160]. Similarly, coral-associated bacteria have been maintained in coral cell-free culture fluid (i.e., sterilized coral homogenates), and the health state of the coral was shown to influence bacterial growth [165]. Intracellular bacteria may be able to be isolated using such a technique, and metabolic analyses of cell-free culture fluids may provide additional insights in the metabolites that are necessary for the growth of intracellular bacteria. Successful culturing of bacterial endosymbionts of corals, based on genomic and transcriptomic data, would open a wide avenue of applications.

## 5. Applications Involving Coral-Associated Intracellular Bacteria to Help Save Coral Reefs

Beyond understanding the fundamental aspects of intracellular symbiosis, host–endosymbiont interactions and co-evolution, mutualistic intracellular symbionts bear great interest in the applied sciences, particularly in the microbiome manipulation field. Microbiome manipulation is one of several avenues that are currently being pursued in order to mitigate coral bleaching [40,53,54,167,168,169,170]. As excess ROS production by Symbiodiniaceae is one of the main causes of coral bleaching [34,36,37,38], it was postulated that treating corals with bacteria with a high ROS-scavenging ability could mitigate coral bleaching [171]. Proof of concept studies have confirmed that the coral microbiome can be modified [172,173], without specifically targeting ROS scavenging, but only three studies so far have actually trialed probiotics to mitigate coral bleaching [174,175,176]. Some of the major potential caveats are as follows: (i) will the bacteria persist in the corals, or should probiotics be applied regularly/during every bleaching event? and (ii) how can the inoculated bacteria be prevented from spreading in the surrounding waters and organisms and potentially disrupting other parts of the ecosystem? Establishing specific associations with vertically transmitted, intracellular bacteria would solve both issues. This approach has been implemented by using *Wolbachia* in the mosquito *Aedes aegypti* to reduce the transmission of arboviruses, including viruses causing dengue, chikungunya, and Zika [177]. *Wolbachia* is an intracellular α-proteobacterium that is estimated to naturally infect 40–60% of insect species [178,179]. Its transmission through host generations is mainly maternal through their eggs, and its dispersion is highly enhanced by several mechanisms that impact host reproduction, with the main one being cytoplasmic incompatibility. Infected females can mate with both uninfected and infected males, leading to *Wolbachia* transmission, but mating between an infected male and an uninfected female, i.e., not resulting in *Wolbachia* transmission, leads to offspring loss [180]. *Wolbachia* was initially reported to provide viral protection to *Drosophila melanogaster* [181] and was subsequently shown to prevent the transmission of a wide range of human arboviruses in *A. aegypti* by inhibiting pathogen replication [182,183,184]. This *Wolbachia* strain had to be stably introduced into *A. aegypti* laboratory populations [184,185], which do not naturally associate with *Wolbachia*. Early fieldwork trials in northern Australia have shown that, following the release of *Wolbachia*-infected mosquitos in the wild, *Wolbachia* has established in the wild uninfected populations, reaching around 90% of the populations [186,187,188]. Furthermore, dengue transmission has significantly decreased in these regions [177,188]. Hence, microbiome manipulation using an intracellular bacterium has been particularly successful in this example and has provided crucial advantages compared to an extracellular bacterium: *Wolbachia* has spread quickly and efficiently in wild populations following a unique release of infected mosquitos and has remained stable over the years; the risk of horizontal transmission to other animals, including humans, is very low [189]. As a long-term solution for coral bleaching mitigation, coral microbiome manipulation with intracellular bacteria should therefore be pursued.

Finally, if intracellular symbionts can be pure cultured, this will open the possibility of genetic engineering aimed at introducing specific traits of interest that might not be naturally present in the intracellular bacterial strain of interest. A recent proof of concept study has shown the possibility of transforming *Spiroplasma poulsonii*, an endosymbiont of *D. melanogaster*, via plasmids [190]. These promising results indicate that commonly used genetic engineering tools can be applied to intracellular bacteria. However, genetic modification of symbionts and reintroduction in a host, also referred to as paratransgenesis, has only been performed with extracellular bacteria. For example, gut symbionts of the triatomine bug *Triatoma infestans*, the vector of the Chagas’ disease pathogen *Trypanosoma cruzi,* were genetically modified to express antimicrobial peptides or antibody fragments, efficiently reducing *T. cruzi* numbers when reintroduced in trypanosome-infected hosts [191,192]. With a growing number of intracellular bacteria being successfully obtained in pure culture, this approach will surely be increasingly used in the coming years. Directed evolution is another approach that may allow for the generation of endosymbionts with enhanced abilities [54] and for which regulatory approval and public acceptance will be less challenging to obtain. Cultured Symbiodiniaceae (*C. goreaui*) were maintained for several years at a high temperature (31 °C, versus 27 °C in normal conditions) and were subsequently shown to have better performance during a thermal stress event, both in culture and *in hospite* [157,193]. A similar approach could be used for pure-cultured bacterial endosymbionts to enhance their ability to favor thermal resilience in a coral host [54].

## 6. Conclusions

While intracellular bacteria have been widely studied in animals, particularly in insects, they remain under-investigated in corals. Great advancements have been made in recent years to understand the bacterial communities associated with corals, across a wide range of host species, developmental stages, and geographical locations. The same efforts should be sustained to specifically explore intracellular bacteria and to overcome the technical challenges that have slowed down this research area. Approaches that have proved fruitful in other systems will guide this research. Our specific recommendations are as follows:

- To characterize and localize stably associated bacteria and bacteria associated with gametes and temporary gonads as a starting point in the search for intracellular and vertically transmitted bacteria;
- To focus research on specific coral microhabitats and structures, such as Symbiodiniaceae and CAMAs to find intracellular bacteria, and to isolate successful microhabitats to perform meta-omics studies; and
- To use bacterial genome data to optimize culture media for attempting to grow intracellular bacteria in pure culture.

Information on the cellular location, transmission mode, taxonomic affiliation, and function of intracellular bacteria will allow us to characterize the most intimate host-bacteria interactions within the coral holobiont. From a fundamental perspective, it will be an opportunity to compare coral–bacteria intracellular interactions with more established systems, such as plants and insects, and to assess whether specific mechanisms are conserved across such divergent taxa. From an applied point of view, successfully cultured intracellular bacteria would make ideal candidates for coral microbiome manipulation to try and mitigate coral bleaching. In particular, if genetic engineering were to be considered, symbionts that are vertically transmitted and that show little to no contact with the surrounding environments would have to be used to avoid a potential spread of genetically modified bacteria beyond the coral host. Endosymbiosis is an exciting and promising field and should not be left aside in cnidarians, especially in the quest to reduce the negative impacts of global climate change on coral reefs.

## Data Availability

Not applicable.
